# Anti-inflammatory effect of resveratrol in human coronary arterial endothelial cells via induction of autophagy: implication for the treatment of Kawasaki disease

**DOI:** 10.1186/s40360-016-0109-2

**Published:** 2017-01-09

**Authors:** Fu-Chen Huang, Ho-Chang Kuo, Ying-Hsien Huang, Hong-Ren Yu, Sung-Chou Li, Hsing-Chun Kuo

**Affiliations:** 1 Department of Pediatrics, Kaohsiung Chang Gung Memorial Hospital and Chang Gung University College of Medicine, Kaohsiung, Taiwan; 2 Kawasaki Disease Center, Kaohsiung Chang Gung Memorial Hospital, Kaohsiung, 833 Taiwan; 3 Genomics and Proteomics Core Laboratory, Department of Medical Research, Kaohsiung Chang Gung Memorial Hospital and Chang Gung University College of Medicine, Kaohsiung, Taiwan; 4 Institute of Nursing and Department of Nursing, Chang Gung University of Science and Technology; Chronic Diseases and Health Promotion Research Center, CGUST, Chiayi County, Taiwan

**Keywords:** Kawasaki disease, Resveratrol, Autophagy, Endothelial cells

## Abstract

**Background:**

Kawasaki disease (KD) is an acute febrile vasculitis in childhood, which is the leading cause of acquired heart disease in children. If untreated, KD can result in coronary aneurysms in 25% of patients, and even under intravenous immunoglobulin (IVIG) treatment, 10–20% of children will have IVIG resistance and increased risk of developing coronary arteritis complication. Additional therapies should be explored to decrease the incidence of coronary artery lesions and improve the prognosis in KD. Autophagy has been reported to play a critical role in a variety of heart diseases. Resveratrol (RSV) confers cardio protection during ischemia and reperfusion in rats via activation of autophagy. Serum TNF-alpha levels are elevated in KD, which might activate the endothelial cells to express intercellular adhesion molecule-1 (ICAM-1), vascular cellular adhesion molecule-1(VCAM-1), inducible nitric oxide synthase (iNOS) and IL-1β.

**Methods:**

Human coronary arterial endothelial cells (HCAECs) were either untreated or treated by TNF-α 10 ng/ml for 2 h in the presence or absence of RSV or autophagy-related protein 16-like 1 (Atg16L1) siRNA. Total RNA was analyzed by real-time quantitative PCR for ICAM-1, VCAM-1, iNOS and IL-1β mRNA expressions. The involvement of autophagy proteins was investigated by Western blot.

**Results:**

Pretreatment with resveratrol significantly inhibited TNF-α-induced ICAM-1, iNOS and IL-1β mRNA expression in HCAECs. Western blot revealed the enhanced autophagy proteins LC3B and Atg16L1 expression by RSV. The suppressive effects of RSV were obviously counteracted by Atg16L1 siRNA.

**Conclusions:**

We demonstrated RSV had anti-inflammatory effects on HCAECs via induction of autophagy. Our results suggest that resveratrol may modulate the inflammatory response of coronary artery in KD and explore the role of autophagy in the pathogenesis and alternative therapy of coronary arterial lesions in KD.

## Background

Kawasaki disease (KD) is the leading cause of acquired heart disease in children in the developed world [1]. Patients who suffer coronary artery damage may develop aneurysms and are at risk of clinical cardiovascular events, and even sudden death [2, 3]. Up to 25% of untreated children will develop permanent damage to the coronary arteries and subsequent coronary artery aneurysm [4, 5]. Even though treatment with intravenous immunoglobulin (IVIG) reduces the development of coronary artery aneurysms, about 5% of KD patients still suffer from this critical complication [4]. Alternative therapies should be explored to decrease the incidence of coronary arteritis complication and improve the outcome in KD.

The etiology of KD is unknown, but epidemiological data of KD strongly suggest involvement of infectious agents in its pathogenesis [6]. Combination of microbial infection, immune response and genetic susceptibility is believed to contribute to the development of KD.

Autophagy represents a homeostatic mechanism for the maintenance of normal cardiovascular function and morphology. Autophagy has been detected in heart failure (HF) due to dilated cardiomyopathy [7], valvular and hypertensive heart disease [8], and ischemic myocardium [9]. It is well established that LPS triggers cardiomyocyte autophagy [10]. Recently, *NOD1* and *NLRP1* genes*,* pattern recognition receptors (PRRs) involved in regulation of autophagy, appeared to be partly involved in the pathogenesis of KD [11]. Resveratrol (RSV), a red wine-derived polyphenolic compound, has been shown to have significant effects in various disease models such as cardioprotection in ischemic heart, diabetes, chemoprevention of cancers, etc. RSV confers cardioprotection during ischemia and reperfusion in rats via activation of autophagy. Thus, control of autophagy by RSV may represent a potential target to treat or prevent development of coronary arterial lesions (CAL) in KD.

Elevated production of inflammatory cytokines in KD patients cause damage to the coronary arteries. Marked elevation of TNF-α in the early stages of KD in both human and mouse model [12]. Serum TNF-alpha levels are elevated in KD, which was supposed to activate the endothelial cells. Then, adhesion molecules such as intercellular adhesion molecule-1 (ICAM-1) and vascular cellular adhesion molecule-1(VCAM-1) are expressed in the endothelial cells, resulting in adherence of leucocytes firmly to endothelial cells [13]. The leucocytes then damage the endothelial cells and cause vasculitis. Enhanced inducible nitric oxide synthase (iNOS) expression and increased generation of nitric oxide metabolites in leukocytes and endothelial cells is associated with the progression of coronary artery lesions in acute KD [14]. A significant increase in the plasma levels of oxidative stress (OS) markers has been found either in acute or in late stages of KD [15, 16].

In this study, we examined if the induction of autophagy by RSV played an anti-inflammatory effects on TNF-alpha-induced expression of adhesion molecule (VCAM-1 and ICAM-1) and production of cytokine (interleukin (IL)-1beta and iNOS) in HCAECs.

## Methods

### Cell culture and treatments

Human coronary arterial endothelial cells (HCAECs) were obtained from the American Type Culture Collection and maintained at 37 °C under humidified 5% CO_2_ in a stationary culture. HCAECs were cultured in Endothelial Cell Growth Kit-BBE (ATCC® PCS-100-040), and used at passages two or three. HCAECs were exposed to 10 ng/ml TNF-α (R&D Systems, Minneapolis, MN, USA) for the indicated times.

### Reagents

Standard laboratory reagents were obtained from Sigma (St. Louis, MO, USA) or Fisher Scientific (Pittsburgh, PA, USA).

### Cell fractionation

Cytosolic, membranous and nuclear extracts from untreated and treated cultured cells were prepared as previously described [17]. Protein concentrations in cell fractions were determined by a Bio-Rad protein assay kit (Bio-Rad Laboratories, Hercules, CA, USA) and normalized before loading for Western blot.

### Western blotting

Equal amounts of total protein were separated by SDS-PAGE and then transferred to nitrocellulose membranes by semi-dry blotting as previously described [18, 19]. After blocking the membranes with 5% non-fat dry milk, they were probed with antibodies to ATG16L1, Beclin-1 and LC3B (Cell Signaling, Beverly, MA) and then developed with HRP-conjugated second antibodies (Zymed Laboratories, San Francisco, CA) and enhanced chemiluminescence (Pierce Chemical Co., Rockford, IL). Capture the chemiluminescent signals using a camera-based imager and use image analysis software to read the band density of the target protein.

### RNA Isolation and cDNA Synthesis

Total RNA was extracted from cultured cells with the Trizol reagent (Invitrogen Corporation, Carlsbad, CA), following the manufacturer’s directions. The RNA was reverse-transcribed with primers using the GeneAmp kit (Roche, Nutley, NJ) as described in detail earlier [20].

### Real-time quantitative reverse transcription PCR

Real-time quantitative reverse transcription-PCR analyses were performed in a fluorescence temperature cycler (LightCycler; Roche Diagnostics) as described previously [20, 21]. The following primers were used: iNOS, 5′- GTTCTCAAGGCACAGGTCTC-3′ (forward primer) and 5′-GCAGGTCACTTATGTCACTTATC-3′ (reverse primer); IL-1β, 5′-GAGCAACAAGTGGTGTTCTCC (forward primer) and AACACGCAGGACAGGTACAG-3′ (reverse primer); ICAM-1, 5′-ACAAGTGCCGTGCCTTTAGCTC-3′ (forward primer) and 5′-GATCACGAAGCCCGCAATG-3′ (reverse primer); VCAM-1, 5′-GGA-TGCCGGAGTATACGAGTGTG-3′ (forward primer) and 5′-CAATGGCGGGTATTACCAAGGA-3′ (reverse primer); and glyceraldehyde-3-phosphate dehydrogenase, 5′-CCAGCCGAGCCACATCGCTC-3′ (forward primer) and 5′-ATGAGCCCCAGCCTTCTCCAT-3′. All quantifications were normalized to the housekeeping gene glyceraldehyde-3-phosphate dehydrogenase. The mRNA expression levels were measured using the comparative threshold cycle (△△Ct) method of relative quantitation.

### RNA interference (RNAi) in cultured cells

RNAi experiments in cultured cells were performed as described previously [17, 22] with some modification. Briefly, subconfluent HCAECs were transfected with chemically synthesized siRNA at a siRNA concentration of 20-40nM using Lipofectamine 2000 (Thermo Fisher Scientific, Waltham, MA, USA) and serum-free medium (OPTI-MEM) according to the manufacturer’s recommendations. After transfection, the cells were treated by RSV, TNF-α or combination of both, and RNA or proteins were extracted for further experiments.

### Cell viability and morphologic features

Representative cell populations from each condition were examined under light microscopy. No significantly morphologic change was observed under any condition. Trypan blue exclusion test was used to confirm the cell viability in untreated or treated cells.

### Statistical analysis

All above experiments were carried out at least in triplicate with similar results. Values were shown as means ± SE. Statistical analysis was performed using the paired Student’s *t*-test and ANOVAs (StatView; SAS Institute, Cary, NC). *P* values < 0.05 were considered significant.

## Results

### Inhibitory effects of RSV on TNF-α-induced ICAM-1 mRNA expression

**Fig. 1:**
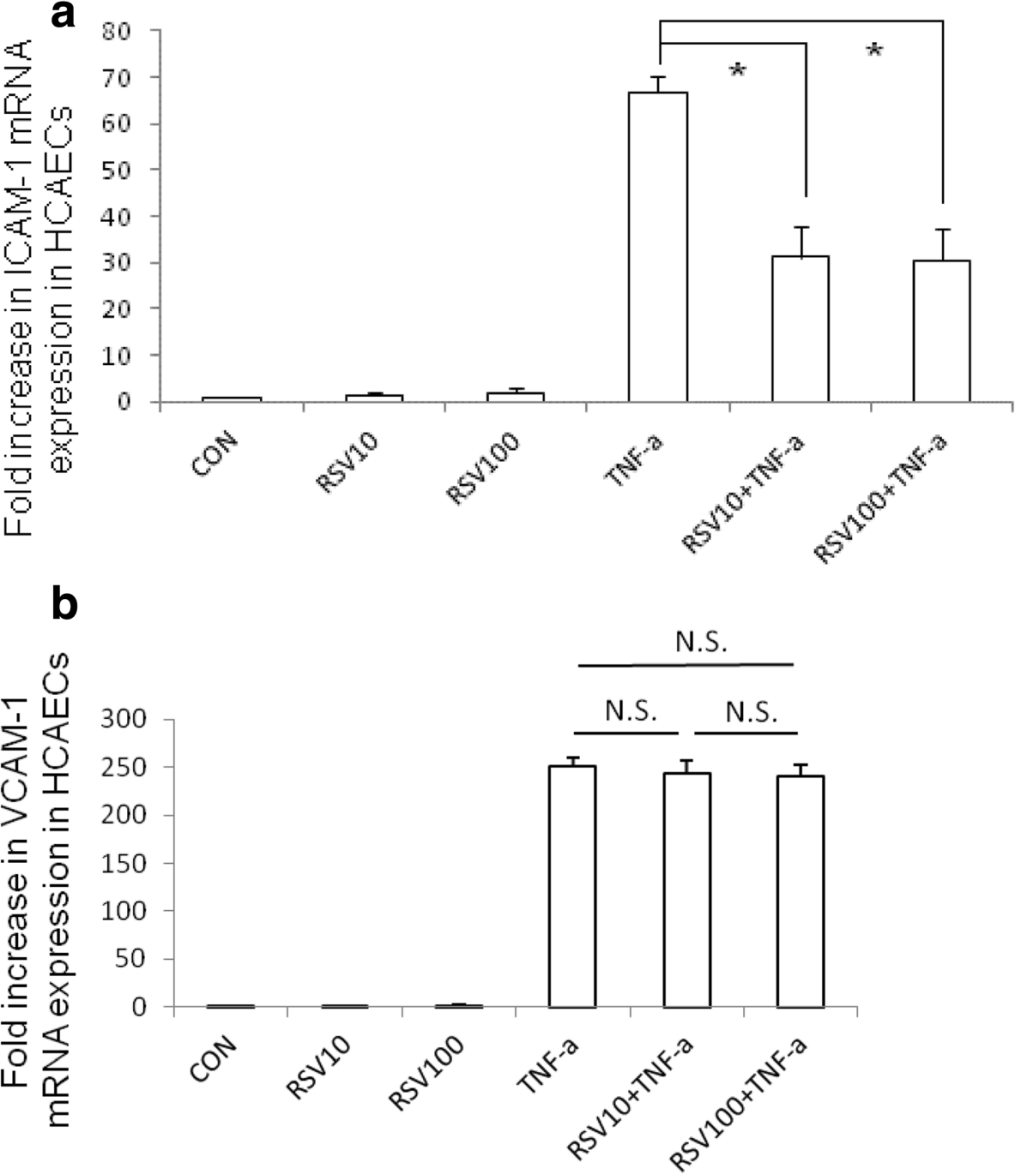
The effect of RSV on TNF-α induced ICAM-1 and VCAM-1 mRNA expression in HCAECs. HCAECs were either untreated (CON) or treated by TNF-α 10 ng/ml for 2 h in the presence or absence of RSV 10 μM or 100 μM (RSV10 and RSV100). Total RNA was prepared after infection and analyzed by real-time quantitative PCR to estimate amounts of ICAM-1 and VCAM-1 transcript. The amounts of ICAM-1 (**a**) and VCAM-1 (**b**) mRNA produced and normalized to the corresponding amount of GAPDH transcript were shown as the fold increase over untreated control cells. Results are represented as means ± S.E.M. for at least three determinations from independent experiments. (*p < 0.05 indicates significance, compared to TNF-α stimulation only)

TNF-α is necessary for induction of coronary artery inflammation and aneurysm formation in an animal model of KD [23]; therefore, we used TNF-α to treat human coronary endothelial cells as an in vitro model for KD. HCAECs were either untreated or treated by TNF-α 10 ng/ml for 2 h in the presence or absence of RSV 10 μM or 100 μM. Total RNA was analyzed by real-time quantitative PCR (RT-PCR) for ICAM-1 and VCAM-1 mRNA expressions. As shown in Fig. 1, TNF-α induced ICAM-1 and VCAM-1 mRNA expression in HCAECs while RSV alone did not induce significant amount of ICAM-1 and VCAM-1 mRNA expression. RSV suppressed TNF-α-induced ICAM-1 mRNA expression but had no significant effect on TNF-α-induced VCAM-1 mRNA expression. However, there was no further suppressed effect on TNF-α-induced ICAM-1 mRNA expression when the dose of RSV was increased to 100 μM.

### The involvement of autophagy on the suppressed effects of RSV on TNF-α-induced ICAM-1 mRNA expression

**Fig. 2:**
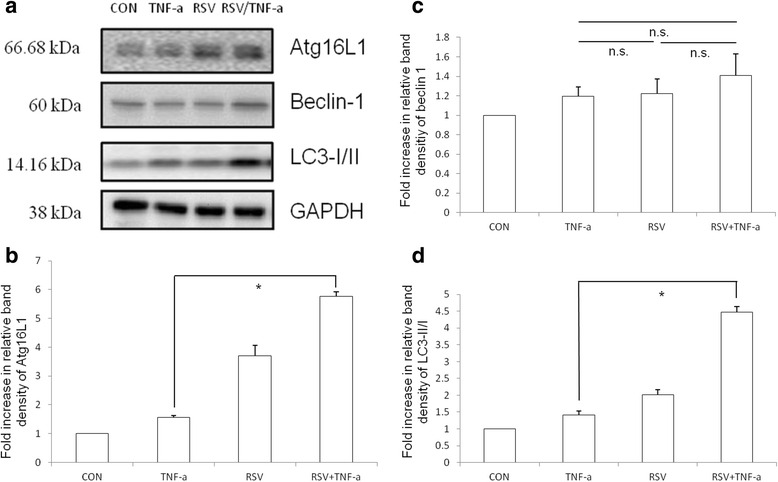
The proteins expression of autophagy in TNF-α-treated HCAECs in the presence of RSV. HCAECs were either untreated (CON) or treated by TNF-α 10 ng/ml for 2 h in the presence or absence of RSV 10 μM (RSV). The Western blots illustrate the expression of Atg16L1, Beclin 1 and LC3B proteins in cytosolic extracts of HCAECs. The results shown are representative of three separate experiments. GAPDH worked as a normalization of cytosolic protein. Representative immunoblots (**a**) and densitometric quantification of immunoreactive bands are shown. The relative band intensities of Atg16L1 (**b**), Beclin 1 (**c**) and LC3-II (**d**) in HCAECs were quantified as fold increases compared with the control cells. Each value represents the mean ± S.E.M. of 3 independent experiments. An asterisk indicates a significant difference (*p* < 0.05)

**Fig. 3:**
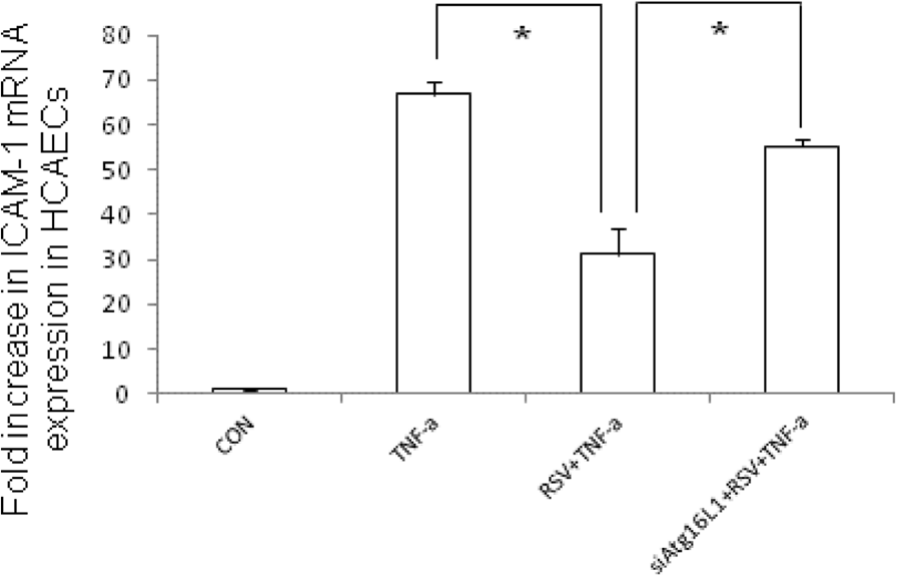
The involvement of Atg16L1 in the negative regulation of RSV on TNF-α induced ICAM-1 mRNA expression in HCAECs. HCAECs were either untreated (CON) or treated by TNF-α 10 ng/ml for 2 h in the presence or absence of RSV 10 μM (RSV) or Atg16L1 siRNA (siAtg16L1). Total RNA was prepared after infection and analyzed by real-time quantitative PCR to estimate amounts of ICAM-1 transcript. The amounts of ICAM-1 mRNA produced and normalized to the corresponding amount of GAPDH transcript were shown as the fold increase over untreated control cells. Results are represented as means ± S.E.M. for at least three determinations from independent experiments. (*p < 0.05 indicates significance, compared to TNF-α stimulation only)

To further evaluate which signaling pathway was involved in regulatory effects of RSV on TNF-α induced ICAM-1 mRNA expression in HCAECs, we investigated the intracellular signal pathways. Based on previous study showing RSV attenuates vascular endothelial inflammation by inducing autophagy [24], we investigated the involvement of autophagy on the negative regulation of RSV on TNF-α induced ICAM-1 mRNA expression in HCAECs. HCAECs were either untreated or treated by TNF-α 10 ng/ml for 2 h in the presence or absence of RSV 10 μM, and the ratio of LC3-II/I, a central marker of autophagy, was analyzed in whole cell protein by Western blot. Western blot data showed that TNF-α induced the expression of the ratio of LC3-II/I, which was significantly upregulated by RSV (Fig. 2), suggesting the involvement of autophagy in the regulatory effect of RSV. Beside LC3II/I‚ Atg16L1, a key protein in the formation of autophagosome, was enhanced by RSV while there was no significant enhancement of Beclin 1. To verify the role of Atg16L1 on the regulatory effects of RSV on TNF-α induced ICAM-1 mRNA expression in HCAECs, after transfected with Atg16L1 siRNA, the cells were either untreated or treated by TNF-α 10 ng/ml for 2 h in the presence or absence of RSV 10 μM. Total RNA was analyzed by real-time quantitative PCR (RT-PCR) for ICAM-1 (Fig. 3) mRNA expressions. We observed the RSV-mediated suppression of TNF-α induced ICAM-1 mRNA expression in HCAECs was abolished in the presence of Atg16L1 siRNA. It suggests the involvement of autophagy protein Atg16L1 in the suppressed effects of RSV on TNF-α-induced ICAM-1 mRNA expression.

### The involvement of Atg16L1 in the inhibitory effects of resveratrol on TNF-α-induced iNOS mRNA expression

**Fig. 4:**
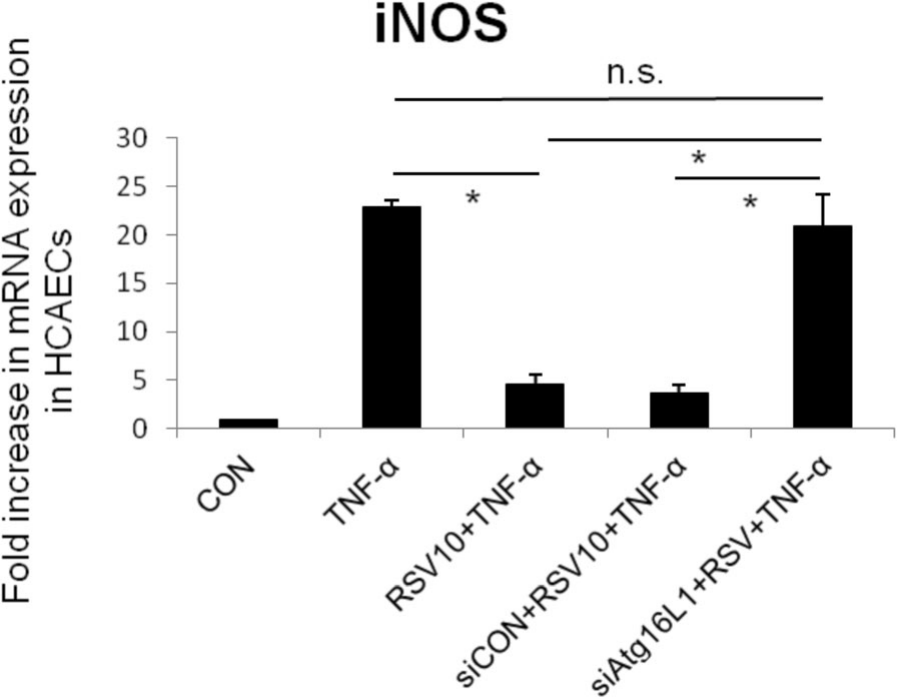
The involvement of Atg16L1 in the negative regulation of RSV on TNF-α induced iNOS mRNA expression in HCAECs. HCAECs were either untreated (CON) or treated by TNF-α 10 ng/ml for 2 h (TNF-α) in the presence or absence of RSV 10 μM (RSV10), control siRNA (siCON) or Atg16L1 siRNA (siAtg16L1). Total RNA was prepared after infection and analyzed by real-time quantitative PCR to estimate amounts of iNOS transcript. The amounts of iNOS mRNA produced and normalized to the corresponding amount of GAPDH transcript were shown as the fold increase over untreated control cells. Results are represented as means ± S.E.M. for at least three determinations from independent experiments. (*p < 0.05 indicates significance, compared to TNF-α stimulation only)

HCAECs were either untreated or treated by TNF-α 10 ng/ml for 2 h in the presence or absence of RSV 10 μM or 100 μM or Atg16L1 siRNA. Total RNA was analyzed by real-time quantitative PCR (RT-PCR) for iNOS mRNA expression. As shown in Fig. 4, 10 μM RSV suppressed TNF-α-induced iNOS mRNA expression but there was no further suppressed effect on TNF-α-induced iNOS mRNA expression when the dose of RSV was increased to 100 μM. This inhibitory effect of RSV was counteracted by Atg16L1 siRNA.

### The involvement of Atg16L1 in the inhibitory effects of resveratrol on TNF-α-induced IL-1β mRNA expression

**Fig. 5:**
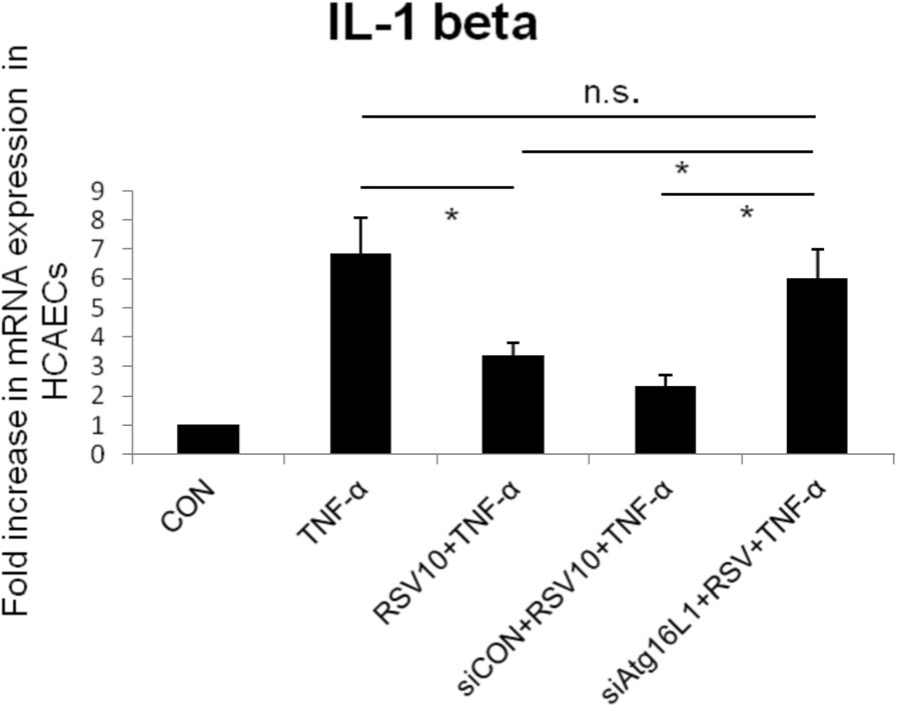
The involvement of Atg16L1 in the negative regulation of RSV on TNF-α induced IL-1β mRNA expression in HCAECs. HCAECs were either untreated (CON) or treated by TNF-α 10 ng/ml for 2 h (TNF-α) in the presence or absence of RSV 10 μM (RSV10), control siRNA (siCON) or Atg16L1 siRNA (siAtg16L1). Total RNA was prepared after infection and analyzed by real-time quantitative PCR to estimate amounts of IL-1β transcript. The amounts of IL-1β mRNA produced and normalized to the corresponding amount of GAPDH transcript were shown as the fold increase over untreated control cells. Results are represented as means ± S.E.M. for at least three determinations from independent experiments. (*p < 0.05 indicates significance, compared to TNF-α stimulation only)

HCAECs were either untreated or treated by TNF-α 10 ng/ml for 2 h in the presence or absence of RSV 10 μM or 100 μM or Atg16L1 siRNA. Total RNA was analyzed by real-time quantitative PCR (RT-PCR) for IL-1β mRNA expression. As shown in Fig. 5, 10 μM RSV suppressed TNF-α-induced IL-1β mRNA expression but there was little further suppressed effect on TNF-α-induced IL-1β mRNA expression when the dose of RSV was increased to 100 μM. This inhibitory effect of RSV was nearly abolished by Atg16L1 siRNA.

## Discussion

Resveratrol is a beneficial pharmacological tool that augments autophagy to bring about reverse remodeling in the post-infarction heart [25]. Resveratrol attenuates vascular endothelial inflammation via activation of autophagy [24]. Up-regulation of endothelial nitric oxide synthase [26] and autophagy-related genes [26, 27] by RSV in human umbilical vein endothelial cells (HUVECs) may protect the cells from oxidative damage [27]. These results indicate the beneficial effects of resveratrol on the cardiovascular system by regulating autophagy. We observed induction of autophagy expressions by RSV inhibited TNF-α-induced ICAM-1, iNOS and IL-1β expressions in HCAECs. It is reasonable to use RSV as an adjuvant therapy for KD patients to enhance their autophagy expression and attenuates coronary endothelial inflammation, which may lead to aneurysm formation.

TNF-α released by activated monocytes, are found in atherosclerotic plaques and in significantly greater levels in abdominal aortic aneurysm [28], which can enhance ICAM-1 expression on endothelial cell cultures in vitro [29]. Recruitment of inflammatory cells by ICAM-1 may result in destruction of aorta wall leading to aneurysm formation [30], suggesting a role for ICAM-1 in the initiation of endothelial dysfunction and aneurysm formation. Actually, ICAM-1 contributes to vascular inflammation and early atherosclerosis [31, 32]. By using HCAECs, we observed RSV downregulated the TNF-α -induced ICAM-1 expression in HCAECs, as demonstrated by another group [33]. Furthermore, we illustrated the involvement of Atg16L1 in the suppressive effect.

Oxidative stress cause endothelial dysfunction and cellular injury that result in vascular damage [34, 35]. Previous studies observed nitric oxide production was significantly higher in patients with KD with coronary artery involvement [15] and decreased after IVIG therapy [16, 36]. Immunohistochemical analysis of the coronary arteries from patients with acute KD revealed enhanced iNOS expression in endothelial cells is associated with the progression of CAL in KD [37]. Spermidine exerts vascular protection by reducing oxidative stress via enhanced arterial expression of autophagy [38]. Both in vivo and in vitro studies showed the protective effects of RSV on vascular endothelial dysfunction by reducing endothelial oxidative stress [39], inhibiting TNFα-induced impairment of nitric oxide bioavailability [40] and preventing reactive oxygen species-induced damage [41]. We demonstrated the involvement of Atg16L1, a key player in the development of the autophagosome, in the suppressed effect of RSV on TNF-α-induced iNOS mRNA expression in HCAECs. It suggests the protective effect of RSV on the coronary endothelial injury in KD via enhanced arterial expression of autophagy.

The involvement of neutrophils in the damage to coronary arteries in acute stage of KD [42] can be explained by the scenario: exposure to bacterial LPS occurs at the onset of KD, LPS-bound neutrophils secrete excess elastase into the circulation [43], and elastase promotes IL-1β secretion from human coronary endothelium [44]. It has generally been assumed that IL-1β is produced predominantly by immune-derived cells. However, in ischemic heart disease patients, atherosclerotic coronary arteries synthesize and express significant IL-1β within the endothelium [45] compared with controls.

The plasma membrane contributes directly to the formation of Atg16L1-positive (LC3-negative) pre-autophagosome precursors [46], which subsequently mature to form autophagosome (LC3-positive). Atg16L1 is one of the additional autophagy-related markers, which can also be used for monitoring autophagy though LC3 has been the most extensively used one [47]. Loss of autophagy protein Atg16L1 promoted the generation of reactive oxygen species and subsequent IL-1β production in response to LPS [48]. We demonstrated the involvement of Atg16L1-mediated autophagy in the suppressive effect of RSV on TNF-α-induced iNOS and IL-1β mRNA expression in HCAECs. Although the link between resveratrol and autophagy is a well-documented fact, the findings about the involvement of previously not described players (Atg16L1) in this anti-inflammatory process in HCAECs is new. However, the mechanisms how RSV enhances Atg16L1 protein expression remain unclear and deserve to be investigated. Altogether, these findings suggest the potentially preventive therapy of RSV on aneurysm formation in KD patients.

## Conclusions

Pretreatment with RSV significantly inhibited TNF-alpha-induced ICAM-1, iNOS and IL-1β expressions in HCAECs via the activation of autophagy. Our results suggest that resveratrol may have anti-inflammatory effects on coronary artery in KD and explore the role of autophagy in the pathogenesis of the CAL and the promising therapy in KD arteritis.

## Abbreviations

ATG16L1: Autophagy-related protein 16-like 1
CAL: Coronary arterial lesions
HCAECs: Human coronary arterial endothelial cells
HUVECs: Human umbilical vascular endothelial cells
ICAM-1: Intercellular adhesion molecule-1
iNOS: Inducible nitric oxide synthase
IVIG: Intravenous immunoglobulin
KD: Kawasaki disease
RSV: Resveratrol
VCAM-1: Vascular cellular adhesion molecule-1
